# Beyond GalNAc! Drug delivery systems comprising complex oligosaccharides for targeted use of nucleic acid therapeutics

**DOI:** 10.1039/d2ra01999j

**Published:** 2022-07-14

**Authors:** Joseph O'Sullivan, Jose Muñoz-Muñoz, Graeme Turnbull, Neil Sim, Stuart Penny, Sterghios Moschos

**Affiliations:** Department of Applied Sciences, Northumbria University Newcastle upon Tyne UK NE1 8ST sterghios.moschos@northumbria.ac.uk; High Force Research Ltd, Bowburn North Industrial Estate Durham UK DH6 5PF

## Abstract

Nucleic Acid Therapeutics (NATs) are establishing a leading role for the management and treatment of genetic diseases following FDA approval of nusinersen, patisiran, and givosiran in the last 5 years, the breakthrough of milasen, with more approvals undoubtedly on the way. Givosiran takes advantage of the known interaction between the hepatocyte specific asialoglycoprotein receptor (ASGPR) and *N*-acetyl galactosamine (GalNAc) ligands to deliver a therapeutic effect, underscoring the value of targeting moieties. In this review, we explore the history of GalNAc as a ligand, and the paradigm it has set for the delivery of NATs through precise targeting to the liver, overcoming common hindrances faced with this type of therapy. We describe various complex oligosaccharides (OSs) and ask what others could be used to target receptors for NAT delivery and the opportunities awaiting exploration of this chemical space.

## Abstract 2

Tapping the glycome space for targeted delivery. We explore GalNAc for targeting oligonucleotides to the liver and ask what other oligosaccharides could expand targeting options for other tissues.

## Graphical abstract

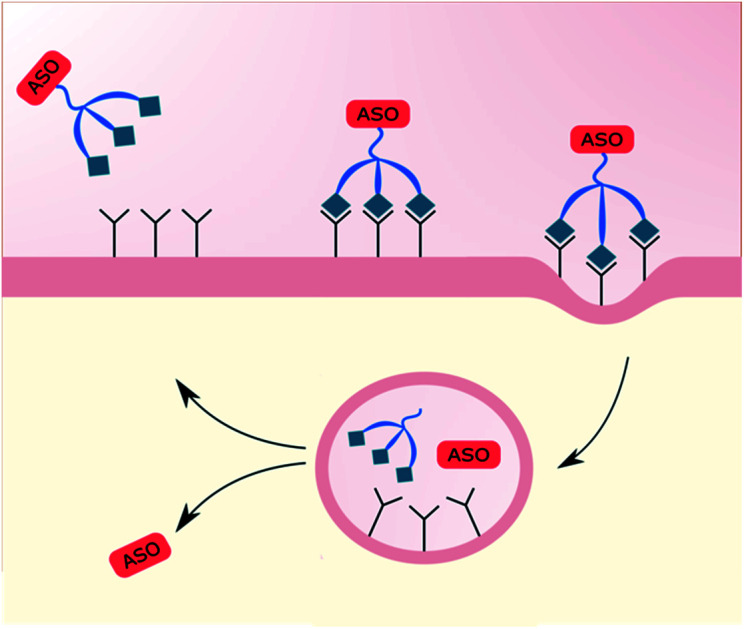

## Introduction

There is undeniably frustration for those with untreatable inherited genetic diseases with a known origin, with current options for some only being that of management, until progression of the disease leads to the patient's death.^1,2^ Where well-characterised genetic changes have been causally linked to heritable disease, treatments can be specifically formulated. For decades the allure of selective gene silencing, quenching, or interference with NATs, and most recently even genome editing, has promised to be the revolutionary jump to a future of precise and personalised therapy for human diseases.^3–5^ There is an attractive quality in the high specificity of these therapies that can limit the materialisation of unwanted and toxic side effects through careful design and meticulous off-target screening.^6^ The routine use of NATs has, however, been impeded. Originally by their susceptibility to degradation from endogenous nucleases, later by the unrecognised finesse of the innate immune response, and consistently with the difficulty these generally negatively-charged macromolecular species experience in crossing cell membranes to reach their target.^7,8^ Persistent research in the field and use of the established interaction of GalNAc with the ASGPR has recently resolved this challenge and enabled enhanced and targeted delivery of functional NATs to the liver, with the ligand–receptor interaction able to facilitate the GalNAc–NAT complex to undergo endocytosis.^9^ This yielded an upsurge in successful clinical trials leading to FDA approvals such as givosiran, a short interfering RNA (siRNA) bound to a triantennary GalNAc ligand (three GalNAc moieties on three precisely spaced branching arms of defined lengths) that assists in the targeted delivery to hepatocytes.^10^ GalNAc-therapeutic conjugates demonstrate the effectiveness NATs can have given a suitable delivery system with specific-targeting glycosides, or glycotargeting, and give hope that similar improvements could be achieved for alternative target cell types or tissues given an appropriately constructed glycosylated delivery system.

## Nucleic acid therapeutics (NATs)

NATs are nucleic acid-based compounds, commonly DNA or RNA in nature but recently oligomers featuring non-natural backbones such as peptide or morpholino chemistries have emerged. These are diverse in therapeutic action and are generally engineered to work by inhibiting, altering the expression, or processing of a gene responsible for a particular disease. Mechanisms of action include endonucleatically active or inactive antisense oligonucleotides (ASOs), antibody-like aptamers, and RNA interference (RNAi) mediators to name a few, which cells can use to tune the outputs of faulty endogenous genes and invasive exogenous genes.^11–13^ Most NATs work through Watson–Crick base pairing to a target transcript or chromosomal locus to produce an effect,^14–16^ opening the possibility for bespoke treatments for specific targets. Sizes range from the shorter locked nucleic acids (LNAs) of approximately 10 nucleotides (nts) (∼3300 Da), containing a synthetic bridge linking the 2′-oxygen with the 4′-carbon of the ribose ring with a minimally modified α-phosphorothioate backbone,^17^ to the larger CRISPR-CAS9 ribonucleoprotein complex involving a bacterially-derived CAS9 protein (∼160 kDa) and a 100 nt guide RNA molecule (∼33 kDa).

In 1978, the concept of the therapeutic use of a nucleic acid, in this case an ASO, was first theorised by Zamecnik and Stephenson whilst working on inhibiting virus replication with oligonucleotides.^18,19^ They suggested that if the target sequence of RNA or DNA was known, a potential future option would be specifically synthesising an oligonucleotide to hybridise to it acting as a virus inhibitor; they also proposed the possibility of hindering protein translation if desired. They then demonstrated how an oligonucleotide 13-mer could prevent translation of certain mRNAs in Rous sarcoma virus. Being aware that nucleic acids are susceptible to degradation, Putney *et al.* found that the use of an α-phosphorothioate nt restricted digestion from exonuclease III.^20^ Additional research led to strategies for further modifications that would improve biostability, boost cellular uptake, and increase potency,^21–23^ including the use of a carrier or drug-delivery system with popular choices being the use of viruses, polymeric nanoparticles, liposomes, and more recently lipid nanoparticle complexes.^24–26^ Some of these currently used modifications are described further by Moschos *et al.*^27^ and Dowdy.^28^ In 1998, success was achieved with the first approval of a NAT for clinical use in fomivirsen, an ASO to treat cytomegalovirus retinitis for patients with AIDS^29^ through an aptamer mechanism of action. In the same year, Fire *et al.* showed that gene silencing could be achieved through use of double-stranded RNA, specifically RNA interference (RNAi), in the nematode worm *Caenorhabditis elegans* (*C. elegans*).^30^ The proceeding years gave the first RNAi-mediated gene silencing in mammalian cells^31^ and the potential of what could be achieved using NATs became apparent. With the field subsequently exploding in popularity over the following twenty years, the goal of reaching the previously thought ‘undruggable space’ was becoming a reality and an expansive range of NATs have made it to market for a variety of ailments with more in clinical trials, demonstrating their versatility in offering more tailored treatments.^32–35^

ASOs were the first class of NATs to hit the market and are generally modified short (20–25 nts) oligonucleotides complimentary to a faulty gene's messenger RNA (mRNA). Nusinersen (a chemically modified phosphorothioate DNA ASO) and eteplirsen (a morpholino phosphorodiamidate ASO) have both recently been approved for treatment of spinal muscular atrophy and Duchenne muscular dystrophy, respectively (see Table 1). Both of these ASOs, and indeed the nusinersen-like *n* of 1 ASO milasen,^36^ function by controlling the splicing of their target genes to overcome gene changes deleterious to the conversion of mRNA into protein.^37^ An alternative format of an ASO is a gapmer, a chemistry involving two runs of modified nucleic acids flanking a DNA oligonucleotide segment; this structure can direct gene silencing through RNase H-cleavage of the mRNA.^37^ In 2018, the FDA approved use of the gapmer inotersen for the treatment of polyneuropathy of hereditary transthyretin-mediated amyloidosis in adults.^38^ Another commonly researched NAT class involves the endogenous gene silencing mechanism of RNAi; a naturally occurring process that consists of RNA, usually 20–25 nts in length, used to silence genes. Therapeutic variations on this theme include double stranded RNA (dsRNA) in the form of custom-designed siRNAs, mimics to the endogenously expressed dsRNA called microRNAs (miRNAs), or single stranded ASOs that are designed to inhibit miRNAs.^39^ A recent approval by the FDA includes patisiran, a siRNA with a lipid-based delivery system^40^ for the treatment of transthyretin-mediated amyloidosis,^41^ with similar systems now also in use in SARS-CoV-2 mRNA vaccines. Aptamers, in part named from the Latin ‘*aptus*’ meaning ‘to fit’, are single stranded oligonucleotides that can be manipulated to bind to specific molecular targets including proteins in a fashion not dissimilar to antibodies, and with comparable *K*_D_'s.^42^ Pegaptanib was the first aptamer approved by the FDA in 2004 for treatment of macular degeneration.^43^ A final example of the diverse range of NATs is clustered regularly interspaced short palindromic repeats (CRISPR). The principle of CRISPR is that of selective gene editing on the genome with an ability to target a malfunctioning gene with a known error and alter that piece of DNA using either a randomly destructive mechanism (non-homologous end joining), or oligonucleotide template-directed repair (homology directed repair). Commonly used alongside CRISPR Associated (CAS) proteins,^16^ the CRISPR-CAS9 system has quickly become the dominant method in the field of gene editing. Unlike other systems, CRISPR-CAS9 can use a single or double stranded oligonucleotide, in addition to the 100 nt guide RNA, to drive homology-directed repair; both oligonucleotides can be modified substantially along the same principles of classical ASO modification to improve stability and prevent innate immunity activation. A notable recent success (2021) has been the use of a CRISPR-CAS9 system for treating sickle cell disease and β-thalassemia, in which patients on the trial responded positively to treatment and no longer endured vaso-occulative episodes.^44^ For additional information, comprehensive reviews on the functions, mechanisms, and delivery of various NATs are available.^7,43,45,46^

**Table 1:** Regulator-approved NATs

Name (market name)	Developer	FDA approval	Modality	Administration	Medical indication	Key references
Fomivirsen (Vitravene)	Ionis	1998	ASO	Intravitreal	Cytomegalovirus retinitis in immunocompromised individuals	Vitravene study Group^52^
Pegaptanib (Macugen)	Valeant	2004	Aptamer	Intravitreal	Macular degeneration	Gragoudas *et al.*^53^
Eteplirsen (Exondys 51)	Sarepta	2016	ASO	Intravenous	Duchenne muscular dystrophy	Mendell *et al.*^54^
Nusinersen (Spinraza)	Ionis	2016	ASO	Intrathecal	Spinal muscular atrophy	Haché *et al.*^55^
Inotersen (Tegsedi)	Akcea	2018	ASO	Subcutaneous	Hereditary transthyretin-mediated amyloidosis	Benson *et al.*^56^
Patisiran (Onpattro)	Alnylam	2018	siRNA	Intravenous	Hereditary transthyretin-mediated amyloidosis	Adams *et al.*^57^
Milasen (N/A)	Boston Children's Hospital	Allowed use 2018	ASO	Intrathecal	Batten disease	Kim *et al.*^36^
Givosiran (Givlaari)	Alnylam	2019	siRNA	Subcutaneous	Acute hepatic porphyria	Balwani *et al.*^58^
Lumasiran (Oxlumo)	Alnylam	2020	siRNA	Subcutaneous	Primary hyperoxaluria type 1	Garrelfs *et al.*^59^
Casimersen (Amondys 45)	Sarepta	2021	ASO	Subcutaneous	Duchenne muscular dystrophy	Shirley^60^

NATs for gene quenching, silencing, or editing have continually been cited as having great potential to herald a new age of personalised therapeutics to treat genetic conditions as well as cancer.^5,47^ Key to the future of NATs is possessing the ability to treat diseases with known genetic origin irrespective of molecular target location in the body, and offer customisable therapies for patients; in some instances, this is already being carried out.^36^ Success to date, however, is driven by first pass metabolism-driven liver loading, leaky cell membranes (Duchenne's muscular dystrophy), and cell uptake after intrathecal injection (spinal muscular atrophy). Crucially, an outstanding and persistent issue that remains is the delivery of the NATs to other target tissues, which must overcome a naturally evolved defence designed to prevent exogenous nucleic acids, such as those being administered, from entering cells.^43,47–49^ ‘Naked’ nucleic acid delivery is therefore challenging due to the susceptibility of these compounds to degradation under physiological conditions from nucleases, non-specific binding, negative charges preventing therapies crossing cell membranes, and inefficient intracellular trafficking and access to cell cytosols.^50,51^ As a result, the development of precise and potent NATs that work exceptionally well in cells has been relatively straight forward in comparison to the problems endured by the challenge posed in delivering them to cells in animals or humans.^47^ With NATs beginning to gain market share, their use is becoming a valid therapeutic option, but there is still a need to improve their stability and delivery *in vivo*, with a hope of perfecting delivery systems to fulfil the promise of these therapies of expanding the druggable space.^61–63^

## GalNAc and the liver

Lectins are carbohydrate-binding proteins with high specificity for particular glycans that can agglutinate cells and aid in the precipitation and removal glycoconjugates.^64,65^ Studies on hepatic lectins, in both animals and humans, presented common findings in their affinity for desilylated molecules possessing a terminal galactose or GalNAc.^66^ Once bound, a process influenced by Ca^2+^ concentration and pH,^67^ the lectin drives endocytosis of the glycosides by liver parenchymal cells and, particularly in the case of glycoproteins, are removed from circulation.^68,69^ In 1980, Baenziger and Maynard suggested there could be a greater binding affinity for terminal GalNAc over galactose when comparing inhibition constants (*k*_i_) calculated through binding assays (up to 27-fold reduction in *k*_i_). A similar reduction in *k*_i_ (∼30-fold) was also observed comparing three galactose residues to two, and four GalNAc residues to two, suggesting binding at multiple sites simultaneously.^70^ This work was furthered by Lee *et al.* showing that branching the glycosides, as well as altering their spacing and flexibility, contributed significantly to the binding (up to 1000-fold).^71^ The preference for these multivalent moieties brought about the term ‘glycoside cluster effect’, which is dependent on two factors: it must contain a lectin with clustered glycan binding sites, and have a multi-valent ligand that can offer the specific glycans to these sites.^72^

**Fig. 1:**
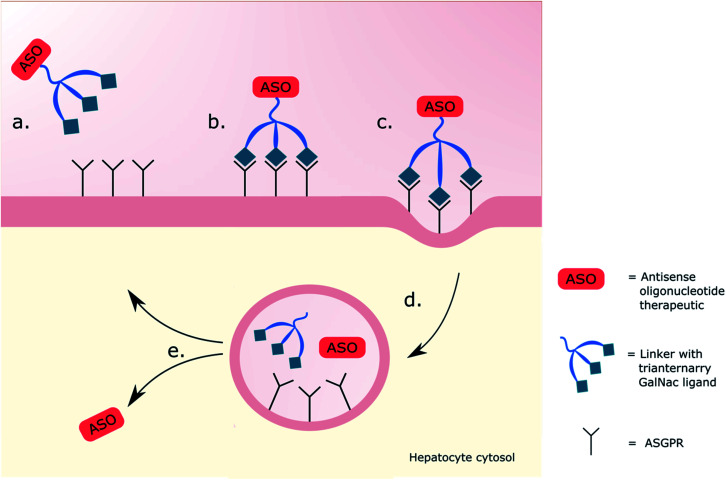
Schematic representation of triantennary GalNAc interaction with ASGPR. (a) A therapy is administered with the intention of targeting hepatocytes. (b) The triantennary GalNAc ligands bind onto three ASGPRs. (c) The GalNAc–receptor interaction initiates clathrin coated pit-mediated endocytosis. (d) Dissociation of ASO from the GalNAc delivery system occurs in the endosome. (e) Endosomal escape of the ASO then allows for release into the cytosol, whilst the ASGPR is recycled to the cell surface.

The ASGPR, a hepatic C-type lectin, presents up to 500 000 copies per cell^73–75^ and is part of a natural trafficking mechanism into liver cells for molecules bearing a terminal galactose or GalNAc.^76^ It thus became an obvious target for enhancing the intracellular delivery of therapeutics aimed at cytosolic hepatocyte targets.^77^ Successful delivery of DNA using a soluble receptor-specific vector through ASGPR was first achieved by Wu and Wu in 1987,^78^ but there was a need to a develop this technology into a safe and reliable system. Promising work was carried out using delivery systems bearing terminal galactose residues,^79,80^ but GalNAc-based systems were favoured along with multivalent systems over singular, previously showing a 3000 and 200 000-fold greater affinity for the ASGPR in competitive binding assays respectively.^70,81–83^ The use of triantennary GalNAc ligands, three GalNAc moieties on three precisely spaced branching arms of defined lengths, noted to be key for the efficacy,^83,84^ became a popular choice of targeting ligand for the ASGPR. Using a ligand of this nature had historically shown promising cellular uptake of oligonucleotide derivative payloads,^85^ with Rensen *et al.* showing a 50-fold increase in affinity for ASGPR in comparison to the triantennary galactose equivalent when delivering glycolipids.^86^ Molecules are attached to the glycoside-based delivery system through the use of a spacer, which itself contributes to the binding affinity of the ligands to the ASGPR; Biessen *et al.* noted that the affinity of a cluster galactoside increased with the spacer length between 4 and 20 Å, with a 20 Å spacer having a 2000-fold higher affinity than a 4 Å one.^87^ Working on many of these principals, Khorev *et al.*^88^ developed a triantennary GalNAc ligand targeting the ASGPR capable of internalising into human parenchymal liver cells carrying fluorescent cargo, visualising endosomes with fluorescence microscopy, and confirming the ASGPR-mediated uptake with asialofetuin in competitive binding assays. They proposed these GalNAc-based ligands held high potential for delivery of therapeutic agents to the liver as, elsewhere, optimisation of the oligonucleotides used in therapeutic delivery were being undertaken by altering the length, backbone charge and stability through phosphorothioate or 2′-*O*-methoxyethyl (MOE) modifications to increase their efficiency.^89,90^ Notable success using therapeutics was limited until 2014 when Prakash *et al.*^75^ reported a breakthrough targeting the ASGPR using a triantennary GalNAc ligand linked through amide bonds to an ASO. This delivery system facilitated endocytosis of the GalNAc–ASO complex improving potency by up to 60-fold in mouse livers. Upon internalisation into the endosome, a drop in pH enables its dissociation from the ASGPR which is recycled to the cell surface.^91^ The therapeutic is lysed from the GalNAc-ligand in the lumen before undergoing endosomal escape, as illustrated in Fig. 1, the mechanism of which is still undetermined.^92^ In the same year, Nair *et al.*^93^ demonstrated dose-dependent gene silencing (ED_50_ 1 mg kg^−1^) in mice using trivalent GalNAc-conjugated siRNA; the novel RNAi offered a 5-fold enhancement compared to the parent design with no adverse effects after sustained dosing. These achievements of robust gene silencing led to the first *in vivo* characterization of the drug disposition, metabolism and toxicokinetics of GalNAc-conjugated therapeutics, showing favourable results, with a 20-fold increase in potency in the knockdown of the hepatic apolipoprotein (a) mRNA when given weekly doses of a GalNAc-conjugated therapeutic (ED_50_ 0.32 mg kg^−1^), in comparison to an unconjugated therapeutic (ED_50_ 6.38 mg kg^−1^), supporting further development in humans.^94^ Clinical trials proceeded with givosiran; a siRNA linked to a trivalent GalNAc ligand that targets delta aminolevulinic acid synthase 1 (ALAS1) mRNA in hepatocytes.^95^ In the phase 1 study, patients with acute intermittent porphyria, inherited disorders in heme biosynthesis, were given monthly or quarterly doses of givosiran (0.035–0.5 mg kg^−1^) or a placebo.^96^ Those provided with monthly doses of givosiran showed consistent and dose dependent reduction in ALAS1 mRNA and the neurotoxic intermediates aminolevulinic acid (ALA) and porphobilinogen (PBG) in urine when compared to the placebo; these reductions correlated with the reduction in neurovisceral attacks. A six-month phase 3 trial similarly showed sustained reductions in ALA and PBG along with a 74% reduction in mean annualized attack rate for patients receiving monthly doses (2.5 mg kg^−1^) of givosiran (*P* < 0.001) in comparison to the placebo. These results led to the first GalNAc-conjugated oligonucleotide therapy to be approved by the FDA in 2019.^58^ Givosiran is marketed for treatment of acute hepatic porphyria and this accomplishment inspired use of GalNAc-based drug delivery systems (DDSs) leading to an array of GalNAc-conjugates currently in varying stages of clinical trials, excellently summarised by Debacker *et al.*^10^ With the approval and commercial success of givosiran and its GalNAc-based DDS, the question arose as to what other glycosides could be used, based on this model, in delivery systems for targeting non-hepatic tissues and cell types.

## Alternative drug delivery

There are several alternative DDSs to the glycan-based receptor-mediated endocytic delivery used in the liver that have been formulated and researched over the previous decades, one of which is viral vectors. Viruses often infect human cells, and the use of virus-based vectors to transport nucleic acids as treatments is exemplified by the 1990 work of Rosenberg *et al.* when they succeeded in gene transfer with the use of retroviruses.^97^ There was initially unease in the use of viral vectors due to issues regarding their toxicity, with caution encouraged, but their safety and efficacy have dramatically improved recently.^98^ Today, viral vectors are indeed popular choices for the treatment of monogenic diseases, the most abundantly used being adeno-associated viruses (AAVs)^99,100^ with notable successes in onasemnogene abeparvovec-xioi.^63^ On the other hand, many in the field still see viral carriers posing risks such as immunogenicity,^16^ and more information on AAV gene therapies may be obtained from other reviews.^101,102^ Nanoparticles (NPs) are typically polymeric structures in the 100 nm diameter range, with variability in shape and size affecting properties including their biodistribution, uptake, and reactivity.^103^ Because of the size and uniform distribution of synthetically engineered NPs (ENPs), they have been seen as a promising solution to achieving NAT delivery.^104,105^ Attempts have been made to address site-specific delivery using targeting ligands on drug loaded NPs for cancer treatments. NPs with monoclonal antibody ligands have been used to target the HER2 in breast cancer cells and glycosylated NPs to target a number of receptors in the liver, breast and lung.^106,107^ Elsewhere, chitosan-modified NPs carrying siRNA showed greater gene silencing in comparison to a lipofectamine control,^108^ demonstrating the power of effective targeting. Another delivery method involves synthetic lipids for DNA transfection (lipofection), but this is an ageing method. Originally thought to be a promising non-viral alternative for the delivery of nucleic acids,^109^ this technique has developed into a modern alternative format through the use of lipid nanoparticles (LNPs) of size distributions similar to polymeric nanoparticles, achieving success in the form of patisiran. LNPs are now more commonplace as they have been specifically developed as a vector for gene therapy,^110^ often targeting immune cells,^111,112^ and use with siRNAs has been successful in silencing particular genes.^113^ Moreover, Moderna Therapeutics and Pfizer/BioNTech recently succeeded in using LNPs as the delivery vector for the approved and globally used mRNA-based vaccines for SARS-CoV-2,^114,115^ and could potentially revolutionise the future of vaccines for many diseases, including therapeutic mRNA delivery for diseases such as cystic fibrosis, where fractional amounts of correctly assembled proteins are considered sufficient for achieving therapy. There remain concerns though, around our continually increasing exposure to NPs such as those from combustion (CNPs) present in diesel exhaust fumes, and ENPs, including TiO_2_ and carbon nanotubes.^116^ It is widely acknowledged that something so small and unpredictable could produce detrimental side effects *in vivo*,^117^ with studies showing NPs frequently producing toxic side effects (nanotoxicity) including inflammation, production of reactive oxygen species (ROS), and apoptosis,^117–119^ particularly when these NPs are inhaled, causing damage to lung cells. Investigations of NP nanotoxicity frequently involve experiments in *C. elegans*, which are easy to cultivate and with a short enough life span to provide generational analysis when exposed to NPs; the advantages and disadvantages of which are well defined by Gonzalez-Moragas *et al.*^120^ Using *C. elegans*, Wu *et al.* demonstrated a correlation between increase in NP dose and ROS production, with the size of these NPs (4–90 nm) behaving differently when treated with antioxidants.^121^ Different exposures to gold and silver NPs exhibited multigenerational nanotoxicity, compounding the unpredictability of this technology.^122,123^ NPs can also induce oxidative stress,^116^ leading to inflammation where they are deposited causing further problems downstream. Because of their small size, NPs can cross the blood–brain barrier^118,124^ and could provide an invaluable method of assisted delivery to the brain, but also run a huge risk of neurotoxicity.^118^ With the lung being another tissue recalcitrant to NAT treatment,^125^ the recent success of LNPs has garnered substantial interest in evaluating their utility for pulmonary drug delivery. However, exposure of rats to In_2_O_3_ ENPs induced problems in the lungs, including pulmonary alveolar proteinosis (PAP), granulomas, and foamy macrophages; it has been therefore advised that the workplace exposure to such NPs is revisited to help protect workers.^126^ More recently, LNP exposure in the context of pre-existing bacterial lipopolysaccharide-induced acute inflammation was demonstrated to exacerbate inflammation in a macrophage- and toll-like immune receptor-dependent means.^127^ Although this response was corticosteroid-responsive, it is unclear how chronic NP dosing, especially in the lung where NP exposure drives foamy macrophage phenotypes, will affect tissue homeostasis beyond the anticipated therapeutic effect. Another common concern is the exposure of expectant parents and their unborn children to conditions with the potential to hinder or alter the foetus during the critical stages of development; it is accordingly proposed that CNPs from air pollution may reach the human placenta.^128^ Overall, the field of NPs needs to be explored further to gain a better understanding of their safety to humans before long term effects of their repeated dosing become unwantedly apparent. It must be emphasised, however, that limited LNP dosage, such as, for example, in the intramuscular administration of a handful of doses of prophylactic vaccines, has not been associated to date with any short or long-term complications in adults, expectant mothers, or foetuses.

Drug delivery systems have made huge strides in the last 10 years, but there are still concerns regarding the safety and ethics of viral vectors including insertional mutagenesis leading to cancer, and the possibility of causing viral treatments to be less efficient.^129,130^ LNPs, after SARS-CoV-2, could conceivably occupy and flourish in the field of vaccinations where infrequent dosing is required. The preference of NPs for dendritic cells and macrophages questions their usefulness for specific alternative targeting and facts around the safety of chronic dosing of NPs still need to be explored, reinforcing the overall need for better and safer targeting systems.

## Complex oligosaccharide-containing delivery systems

Carbohydrates are of biological importance and naturally occurring examples are used in an array of cellular functions in which specific carbohydrate moieties are required.^131^ The ongoing success of the GalNAc-based DDSs for oligonucleotides is fuelling research into the exploration of other glycoconjugates as a potential DDS (or likewise) for numerous targets.^107,132^ The field of glycan science is vast, primarily due to their diversity and structural complexities in architecture: choosing, building, and isolating a chosen OS for one target can be challenging. On the other hand, complex natural OSs are limited in number and there are difficulties in isolating pure products from their sources, particularly where these might be derived from endangered or pathogenic species, and with issues such as homogeneity decreasing their reliability.^133^ If obtaining from a natural source is not a viable option, the assembly of an OS by chemical synthesis, glycoenzymatic biocatalysis, or a combination of the two are popular methods of achieving sufficient, scalable quantities.^134^

**Scheme 1:**
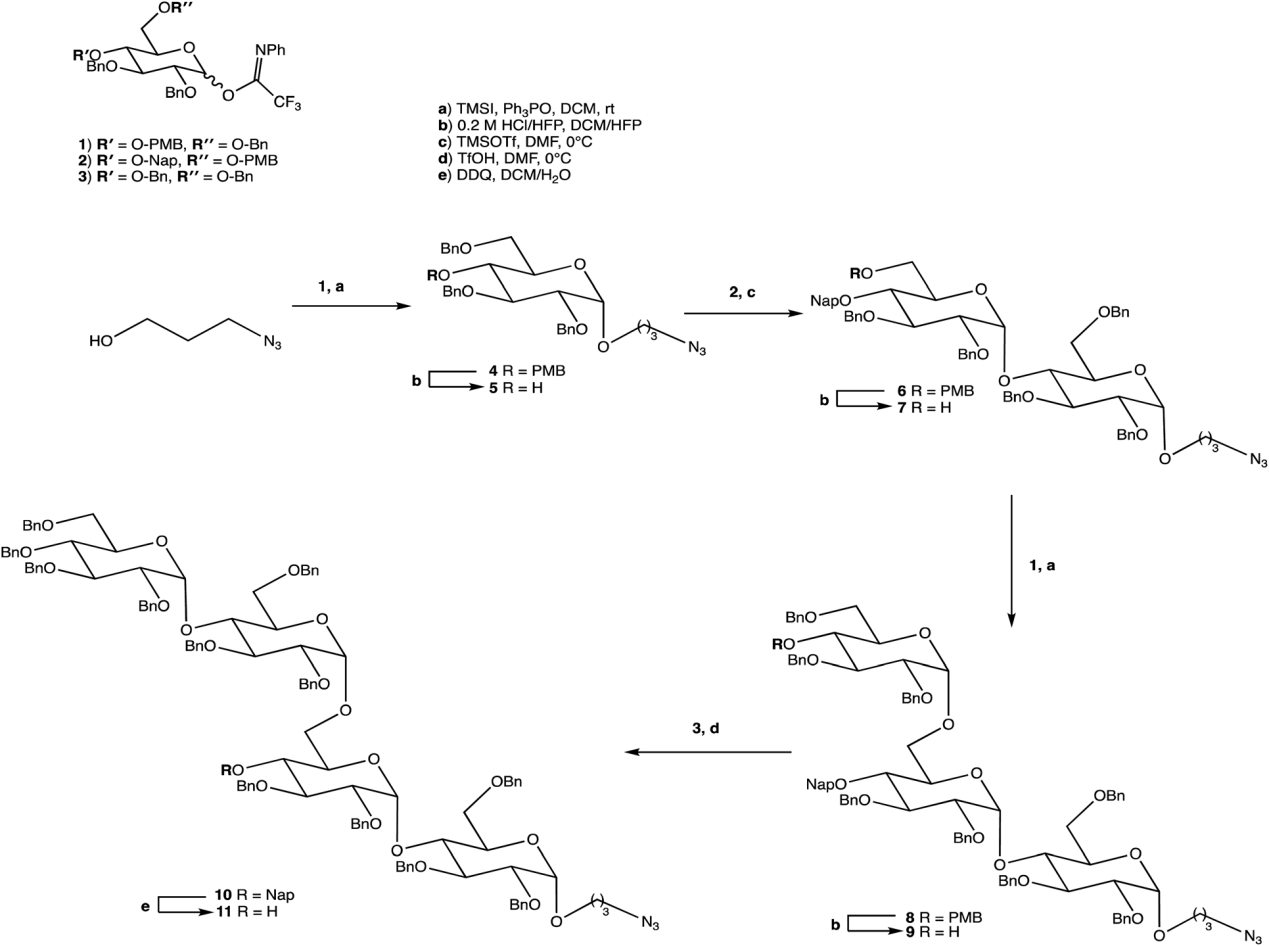
Synthesis of OS 11.

**Scheme 2:**
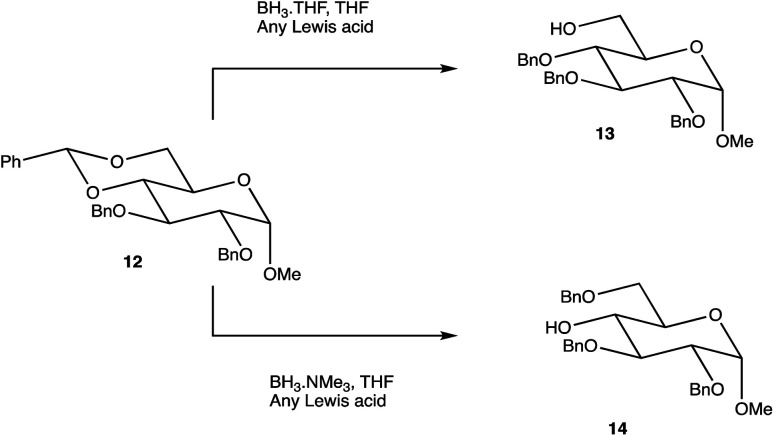
Selective benzylidene ring opening dependent on the borane ligand.

**Scheme 3:**
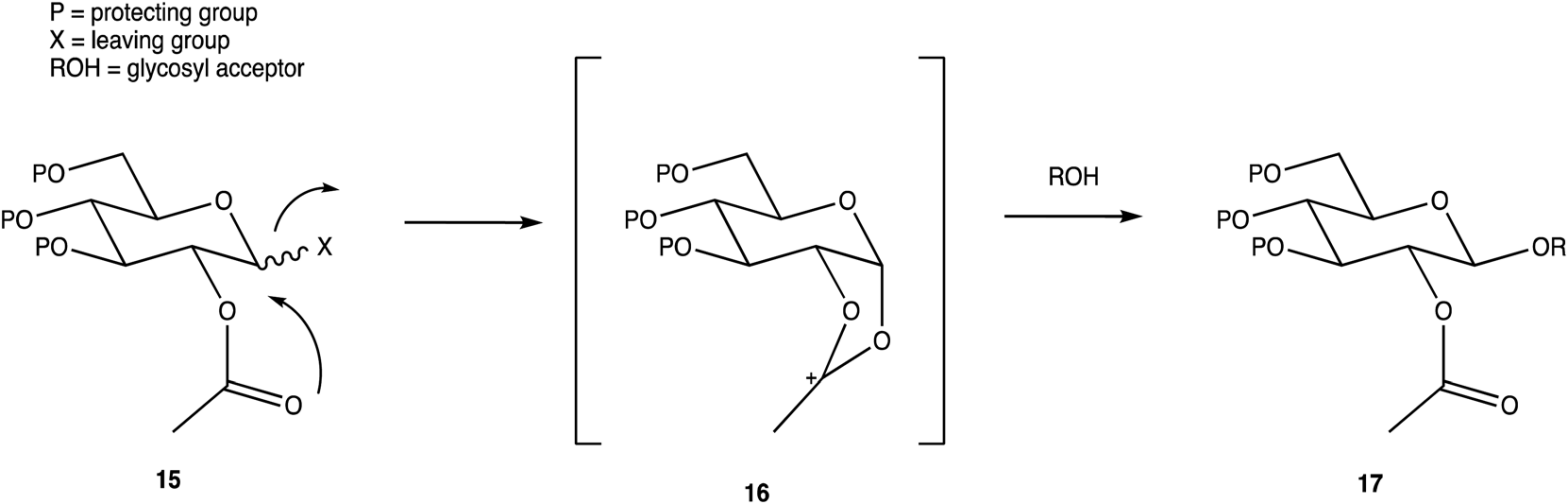
Stereoselective glycosylation with influence from a C2 acyl neighbouring group.

Carbohydrate chemistry in general is an established field, with the first glycosylations occurring in the late 19th century by Michael and Fischer, whose initial work influenced Koenigs and Knorr in the development of glycosylating agents; combined, their work provides the foundations for our current day knowledge.^135,136^ Our understanding of carbohydrate synthesis has vastly increased hence, it is the mutual goal of many to form isomerically-pure glycosides and glycoconjugates to explore biological processes.^137–140^ An attractive feature of using carbohydrates is the unique ability to theoretically assemble a complex of any size and orientation, using only the necessary monosaccharides to build up the desired molecule, giving an array of possible variations for a range of applications. Due to the complexity of OS synthesis, many possible side products may also be obtained, and stereoselective glycosylation in the production of complex OSs has been, and still is, an obstacle for glycomics.^136,141–143^ The glycosidic linkage is commonly formed through use of an electrophilic *glycosyl donor* with a suitable leaving group, a nucleophilic *glycosyl acceptor*, and assisted with help of a *promotor*. Ample research has been carried out in these areas, particularly on the glycosyl donors, from the earlier years with Fischer's work on thioglycosides, Koenigs and Knorr with glycosyl halides,^135,136^ and to the more recent trichloroacetimidates.^144,145^ A multitude of glycosyl donors have also been and are currently being investigated.^141,146^ With the potential to assemble any glycoside in any conformation, complexities arise in controlling the position of the glycosidic bond and the stereochemistry of the resulting product. Generally, each step that increases the size of an OS increases the difficulty in making it due to the possibility of further stereoisomers being formed. Isolating one particular form of an OS to suit the intended target often involves arduous and complex sugar protection strategies (SPSs) with difficulties in separating isomers.^147^ With a substantial body of literature available on how glycosyl donors, promotors, and catalysts can affect the stereospecificity of glycosylation reactions,^148–150^ there are now suggestions to consider how the molar equivalents, reaction temperature, and reaction solvent could also influence this,^151^ which will ultimately be a useful tool in achieving stereospecific glycosylations. The protecting group strategy for building a chosen OS also plays a vital role in concise synthesis, with the priority of hydroxyl groups and orthogonality of the protecting groups key. A popular option is to direct the centre of reactivity through the anomeric carbon with specific activating groups and the protection of all reactive groups before the selective deprotection of the acceptor prior to glycosylation, as shown in Scheme 1. In this partial synthesis, taken from Wang *et al.*,^152^ a protected glycoside is selectively deprotected before it is coupled to another selectively protected monosaccharide with a suitable leaving group. This scheme uses only protected glucosides but shows the varying complexities that must be endured; it is inevitable that using different monosaccharides will affect the yield of isomerically pure complex OSs. An example of a selective deprotection strategy using glucosides is through the use of benzylidene acetal chemistry, explored by Johnsson *et al.*,^153^ shown in Scheme 2. This work shows that the same protected glucoside (12) can undergo selective ring opening to yield the preferred 6′-OH (13) or 4′-OH (14), with the other remaining protected by a benzyl group, and that the regioselectivity is not dependent on the Lewis acid used, but on the type of borane ligand.^153,154^ The influence on selectivity using specific protecting groups must also be considered, one aspect of this being participating protecting groups, and in particular neighbouring-group participation. A recognised example from Guo and Ye involves the formation of 1,2-*trans* glycosides through use acyl groups in the C2 position.^142,155^ As shown in Scheme 3, the C2 acyl group promotes departure of the leaving group (X), forming an intermediate dioxolenium ion (16), leaving only the back side open to attack from a glycosyl acceptor to form only the stereospecific b-glycoside.^146^ Influences such as this must be considered when developing individual synthetic protection strategies to yield isomerically pure OSs and has been the pursuit of many researchers.

**Fig. 2:**
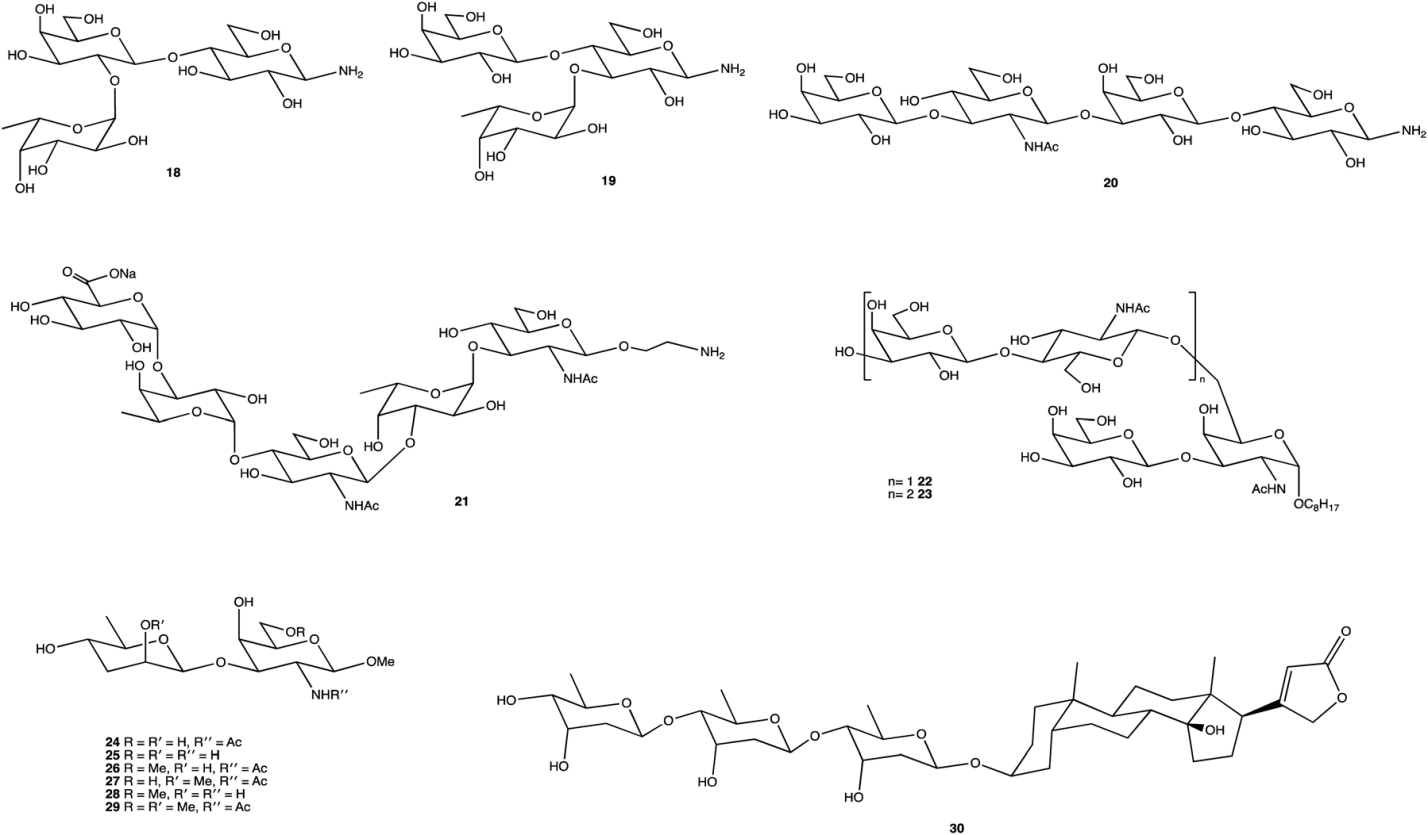
Structures of chemically synthesised glycosides 18–30.

Fig. 2 displays several glycosides (18–30) synthesised using unique SPSs, produced for an array of purposes. Human milk oligosaccharides (HMOs) are a popular area of research and derivatives were constructed by Moore *et al.*, specifically on β-amino HMOs (18–20), to assess their ability to inhibit bacterial growth.^156^ Mandal and Chheda were able to provide a method of synthetically obtaining 21, which is difficult to isolate from natural sources, in sufficient quantities for immunochemical studies.^157^ The human body's interaction with glycosides is well documented, and the synthesis of such, and their derivatives, is frequently carried out, whether to study an OSs interaction with proteins,^158^ to determine enzyme kinetics (22, 23),^159^ or as Cui *et al.* showed, to chemically map an antibody binding site through chemical modification of the terminal disaccharide epitope (24–29).^160^ OS-based therapies are already being used as treatments; the cardiac glycoside digitoxin (30), comprising of a steroidal core with a linked trisaccharide which for over 2 centuries has been used to treat heart failure,^161–163^ and the synthesis of its analogues has been explored for anticancer properties, and heparin, an anticoagulant whose mimetics can play important roles in inflammation, cancer treatments and management of sepsis.^164–166^

The synthesis of clean, isomerically pure OSs is vital to ensuring the reliability of research, with an overwhelming number of factors to consider including reagents, exclusive SPSs, and purification methods. With over a hundred years of chemical glycosylations documented, the harsher conditions used historically are unfavourable to some protection strategies such as those involving acid-labile groups, but with peaking interest in glycosides over the previous year's, thankfully better and greener methods are being developed^167,168^ and will continue to be produced until the field of glycoscience has been sufficiently explored.

**Scheme 4:**
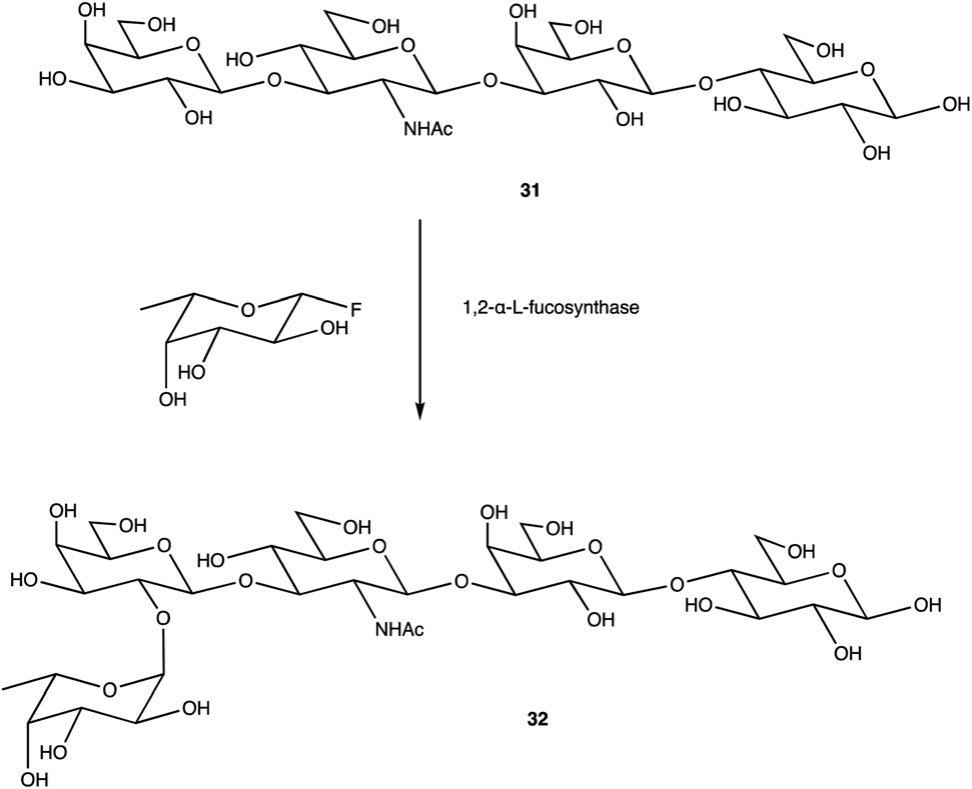
Fucosylation of lacto-*N*-tetraose using a 1,2-α-l-fucosynthase to yield desired terminal disaccharide.

**Scheme 5:**
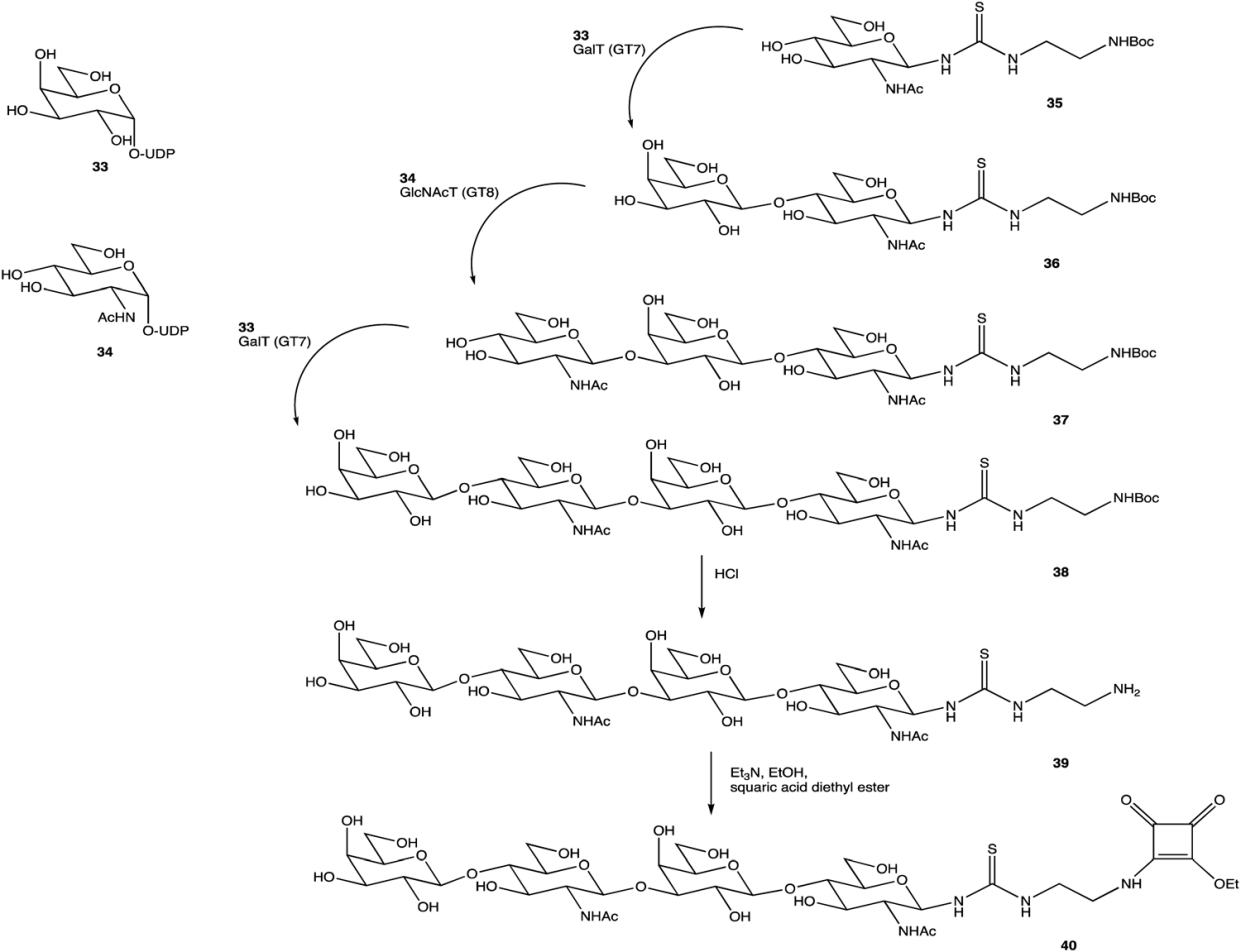
Assembly of OS 38 by consecutive glycosylation with GTs, before chemical synthesis to obtain 40.

**Scheme 6:**
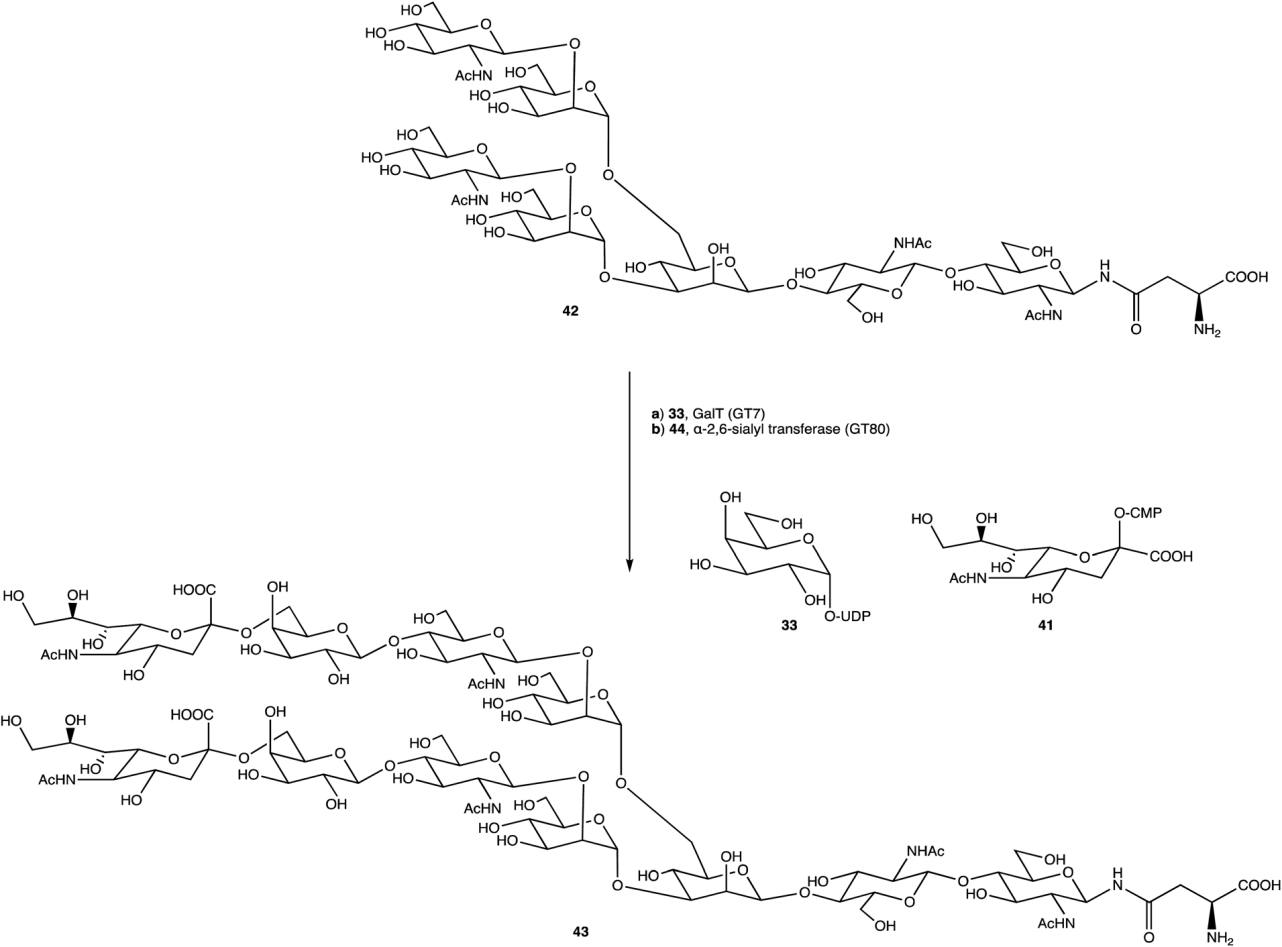
Dodecamer 43 formed through glycosylations with GT7 and GT80.

An alternative option for OS assembly is through the use of glycosyltransferases (GTs); an enzymatic family abundant in nature that can provide regio- and stereo-specific glycosylations for their chosen activities.^169,170^ GTs are classified into families according to sequence homology and structure, which are highly conserved within the family along with its mechanism, but can hold enzymatic activities for different glycosyl donor and acceptors.^131,171^ A wealth of information has been acquired on GTs over the last few decades, but much is still unknown,^172^ with the most recent advances published within the Carbohydrate-Active enZYmes database (https://www.cazy.org) including information on families, structures, and activities of GTs – a set of 114 families currently (February 2022). Use of these enzymes is arguably favourable to the chemosynthetic route due to the reactions being easier, cleaner, and greener given the milder conditions used.^173^ The cloning and expression of enzymes from a variety of sources is well established, but problems with the stability, expression level, and solubility of GTs in aqueous solvents exist due to these enzymes being predominantly membrane-bound which limits their functionality and use in this field. It is possible to alleviate the issue of solubility or produce soluble subunits containing the enzymatically active site through codon optimisation,^174,175^ the use of molecular chaperones, or to obtain the holoenzyme through use of specific or expensive detergents to disrupt the bacterial membrane thereby releasing the enzyme, hopefully in a bioactive state.^176^ Sugiyama *et al.* were successful in producing a glycosynthase, a mutant glycosidase capable of transglycosylation using inexpensive donors (Scheme 4).^177,178^ Through codon optimisation, inversion of a fucosidase to a fucosynthase allowed for fucosylation of lacto-*N*-tetraose 31 producing branched OS 32 containing a terminal disaccharide (fuc-(α)-1,2gal) present in blood H-antigens. They showed that the synthase could also introduce the H-antigen onto a complete glycoprotein, demonstrating the significance of a highly regioselective protein in glycobiology. From the protected glycoside 35, Böcker *et al.* were able to produce the tetrasaccharide 38 through sequential glycosylations; using a β-1,4-galactosyltransferase (GalT), a GT from the family 7 (GT7), and 33 for the addition of galactose, and a β-1,3-*N*-acetyl-glucosaminyltransferase (GlcNAcT) (GT8) and 34 for the addition of GlcNAc (Scheme 5).^179^ These were the limiting steps biocatalytically and chemical synthesis was necessary for deprotection of 38 to 39, and subsequent reaction of the amino group to give tetrasaccharide 40, highlighting the symbiosis of these two fields to yield desired compounds; 40 was subsequently coupled to BSA and its binding affinity for the human galactin-3, a galactose specific lectin, was performed. In contrast, Unverzagt highlights possible complications with chemical synthesis whilst constructing dodecamer 43, as shown in Scheme 6, in which the final stage is performed using GTs. With the objective to integrate 43 into glycopeptides, getting from heptasaccharide-asparagine conjugate 42 chemically would involve complicated SPSs for both the OS and the peptide. The use of specific GTs and reagents, GT7 with 33 followed by GT80 with 41, made it easier and cleaner and again emphasises the benefits of combined chemoenzymatic synthesis

There is a known historical problem of expressing GTs due the hydrophobic nature of them and isolating them from the membrane may require further purification increasing costs, affecting yields, and can ultimately fail to produce a bioactive enzyme or a yield that is economically viable for processes other than basic research. Work needs to be done in this area to develop new routes to help express and solubilise these proteins with assistance from artificial intelligence algorithms and other predictive expression tools, or else provide an alternative or better method for delivering functioning membrane-bound proteins. This would give access to a range of GTs for biocatalytic purposes, opening the field and make it possible to explore this OS space further without limitations. An ability to obtain more GTs could be one key aspect to opening this research, allowing us to create clean and uniquely glycosylated systems for a range of uses, potentially even at the keystroke level of simplicity inherent to modern ASO synthesis and research.

One field that can aid in the exploration of complex OS assembly is that of automated oligosaccharide synthesis (AOSS). Automated synthesis is an established field for both peptides and nucleotides and has enhanced research in these areas by making precise oligomers widely available and highly affordable,^180^ yet despite two decades of oligopeptide and oligonucleotide automated synthesis, success in the OS field, although desirable, has been limited. Two popular methods of chemical OS assembly include the use of resins or polymers as a solid supports attached to the glycosyl donor or acceptor, as first described by Schuerch and Frechet to synthesise a trisaccharide.^181^ Using an automated solid-phase approach, Seeberger *et al.* succeeded in constructing a 50-mer of repeating units, a polymannoside,^182^ and other approaches have been extensively reviewed by Seeberger and Haase,^144^ Castagner and Seeberger,^180^ and Bennett.^183^ The second method is one pot synthesis which can be successful, as demonstrated by Xiao *et al.* in 2020.^184^ For automated one pot synthesis, programmes assist in a streamlined assembly of an OS using designed ‘building block’ monosaccharides without purification of intermediates.^165,185^ The increasing requirement for unique anomerically pure products will likely see a growing interest in the more specific automated enzymatic synthesis of OSs which is still limited by the restricted access to enzymes along with stability issues;^186^ it would be prudent to assume that if more stable GTs were expressed, there could be huge advancement in an enzyme driven AOSS. In depth reviews discussing the chemical and enzymatic AOSS space are well summarised by Panza *et al.*^187^ and Wen *et al.*^188^ respectively.

Commercial AOSS is also beginning to become available with The Glyconeer® (https://glycouniverse.com/glyconeer), a system which uses fully or partially protected monosaccharides along with solid phase resins to build OSs on request. Its effectiveness has been demonstrated by Seeberger *et al.*^189^ The whole system is designed to operate under argon gas to reduce potential hydrolytic side reactions, reaction temperatures can be controlled, and with the smart use of inbuilt UV detectors, products can be monitored. Limitations with this instrument do exist; there are few linkers and resins, and currently only six distinct monosaccharides available to use, although these do cover most conventional glycan building blocks. This instrument is a huge step in the right direction, however one capable of harmonious use of chemical and biocatalytic reagents could catalyse any complex OS synthesis and help develop the field of glycomics.

## Conclusion

Historically, the naked delivery of oligonucleotides hasn't proven successful either experimentally or commercially, but hepatic delivery of NATs has been solved with the success of the GalNAc-based DDS. With the precedent GalNAc has set for NAT delivery, precise targeting with alternative complex OS-based DDSs elsewhere, allowing the distribution of therapeutics to other specified target tissues and tissue-specific cell types is warranted. The recent global adoption of LNPs and mRNA-based therapeutics emphasises the need for better targeting outside the vaccine and hepatic space, and GalNAc has opened the door to demonstrating the kinds of benefits that can be achieved. The huge potential promised for NATs is now being realised, and we speculate that if the paradigm set out by GalNAc is achievable for alternative complex OSs beyond GalNAc, the ability to provide bespoke OS-based DDSs will allow delivery of tailored NATs to targets both *in vitro* and *in vivo*, allowing sugars to play a significant role in this sweet revolution.

## Conflicts of interest

There are no conflicts of interest to declare.

## Supplementary Material
